# A defect in early myogenesis causes Otitis media in two mouse models of 22q11.2 Deletion Syndrome

**DOI:** 10.1093/hmg/ddu604

**Published:** 2014-12-01

**Authors:** Jennifer C. Fuchs, Jennifer F. Linden, Antonio Baldini, Abigail S. Tucker

**Affiliations:** 1 Department of Craniofacial Development and Stem Cell Biology, King's College London, LondonSE1 9RT, UK,; 2 Ear Institute and Department of Neuroscience, Physiology and Pharmacology, University College London, LondonWC1E 8XX, UK; 3 Institute of Genetics and Biophysics, National Research Council, and Department of Molecular Medicine and Medical Biotechnology, University Federico II, Naples80138, Italy

## Abstract

Otitis media (OM), the inflammation of the middle ear, is the most common disease and cause for surgery in infants worldwide. Chronic Otitis media with effusion (OME) often leads to conductive hearing loss and is a common feature of a number of craniofacial syndromes, such as 22q11.2 Deletion Syndrome (22q11.2DS). OM is more common in children because the more horizontal position of the Eustachian tube (ET) in infants limits or delays clearance of middle ear effusions. Some mouse models with OM have shown alterations in the morphology and angle of the ET. Here, we present a novel mechanism in which OM is caused not by a defect in the ET itself but in the muscles that control its function. Our results show that in two mouse models of 22q11.2DS (*Df1/+* and *Tbx1^+/−^*) presenting with bi- or unilateral OME, the fourth pharyngeal arch-derived levator veli palatini muscles were hypoplastic, which was associated with an earlier altered pattern of *MyoD* expression. Importantly, in mice with unilateral OME, the side with the inflammation was associated with significantly smaller muscles than the contralateral unaffected ear. Functional tests examining ET patency confirmed a reduced clearing ability in the heterozygous mice. Our findings are also of clinical relevance as targeting hypoplastic muscles might present a novel preventative measure for reducing the high rates of OM in 22q11.2DS patients.

## Introduction

DiGeorge syndrome, conotruncal anomaly face syndrome and Velo-Cardio-Facial Syndrome are caused by a deletion of a 1.5- to 3 Mb fragment of human chromosome 22q11.2 and as such are commonly referred to as 22q11.2 Deletion Syndrome (22q11.2DS, OMIM #188400) (1). 22q11.2DS is the most common congenital microdeletion syndrome in humans, occurring at a frequency of 1 in 4000 live births, with 5–10% of cases inherited (2,3). Most patients (95%) present with an interstitial deletion encompassing 3 Mb, known as the typically deleted region in which ∼30 genes have been mapped (4). However, smaller deletions (1.5–2 Mb) and unbalanced translocations between chromosome 15 and 22 have also been identified as causing 22q11.2DS manifestations (5,6).

22q11.2DS patients suffer from a wide and phenotypically varied spectrum of anomalies, including palatal anomalies (69–100%) such as submucosal and hard palate clefts (3), velopharyngeal insufficiency, thymus gland hypoplasia, parathyroid abnormalities, malformations of the cardiovascular system and skeletal muscle hypotonia (7). All these malformations can be attributed to developmental defects of the pharyngeal apparatus (3,8–10). Interestingly, the condition is also associated with high frequencies (80–100%) of neurocognitive disabilities (11) and hearing impairment (12).

22q11.2DS patients frequently display conductive (45–84%), sensorineural (11–15%) or mixed (5%) hearing loss, with conductive hearing loss mainly being attributed to inflammation of the middle ear cavity, known as Otitis media (OM) (12). Hearing impairment from early ages can alter behaviour, interfere with the process of speech and language development and lead to learning disabilities. Therefore, early audiological assessment of 22q11.2DS patients and therapy for recurrent episodes of OM is highly recommended (13–15).

OM, in general, is the most common disease in young children worldwide, occurring at least once before the age of 2 in 90% of infants in the developed world (16). The disease is typically classified as either acute or chronic. Acute OM is commonly associated with a viral or bacterial infection and often resolves spontaneously (17). Otitis media with effusion (OME) is characterized by hyperplasia of the middle ear mucosa, excessive serous or mucoid secretions that accumulate in the MEC. In chronic forms of OME, the effusion is infiltrated by inflammatory cells (18). Excessive exudate often leads to conductive hearing loss by the obstruction of middle ear ossicle movement. In severe cases of the disease, OM can be accompanied by tympanic membrane perforation (chronic suppurative OM) and permanent conductive hearing loss owing to ossicle remodelling (19,20). More than 50% of 22q11.2DS patients under the age of 7 display recurrent episodes of OM (21,22). Risk factors for OM are various, including bacterial infections, altered immune status, exposure to tobacco smoke, poor mucociliary clearance and craniofacial abnormalities such as cleft palate and Eustachian tube (ET) dysfunction (22–25). The ET connects the middle ear cavity to the nasopharynx and normally functions to clear secretions from the ear and to equalize middle ear pressure (26,27). Predisposing factors for OM include either a persistently open (patent) or closed ET, variation in length and more horizontal angles, as observed in children under the age of 2, as well as blockage or altered positions of the ET, either provoked by abnormal tissue growth or craniofacial defects such as cleft palates (28–31). In addition to the multifactorial pathogenesis of OM, a role for genetic predisposition is increasingly recognized (32).

As human approaches are limited, murine animal models have been engineered to study the diverse pathobiology of 22q11.2DS. One of these mouse models is the hemizygous *Df1* (deficiency 1)-knockout mouse (*Df1/+*), which carries a multigene deletion in a region of mouse chromosome 16 that is homologous to the 22q11.2 region in humans (33,34). Although the region is conserved, several ancestral rearrangements have led to changes in gene order, thereby shortening the targeted segment, resulting in a deletion of 18 protein-encoding genes that are also deleted in human 22q11.2DS. Despite the deletions not being completely identical, the *Df1/+* mice have been found to recapitulate cognitive and behavioural abnormalities (35,36) and share developmental defects featured in human 22q11.2DS such as cardiovascular abnormalities, although no thymic or gross skeletal abnormalities such as cleft palate have been observed (37). Importantly, these mice also suffer from chronic OM at high incidence, leading to hearing loss (38), and as such are excellent models for studying the mechanisms behind the increased genetic susceptibility to ear disease in 22q11.2DS patients.

The chromosomally engineered *Df1/+* mouse is therefore a good model for the human syndrome and provided the opportunity for gene targeting and transgenic complementation experiments. Such complementation studies using bacterial artificial chromosome mice overexpressing human transgenes have narrowed down the candidate genes within the multigene deletion and highlighted the importance of the T-box transcription factor 1 (*Tbx1*) (39). Heterozygous loss of *Tbx1* results in major structural abnormalities of the heart similar to those observed in *Df1/+* mice (39,40), and chronic OM has been reported (41). The susceptibility to ear disease in 22q11.2DS patients is therefore likely to be specifically caused by the reduced expression of *Tbx1*.

Owing to the high incidence and impact of OM in children globally a number of genetically modified mouse mutants have been identified to unravel the genetic factors that drive the development of OM. The use of mouse models to study the pathobiology of OM has been well established in recent years. Mice that display a chronic inflammation can be categorized as syndromic, non-syndromic, phenotype- or genotype-driven mouse mutants (42). The pathogenesis of OM is multifactorial in mouse mutants, including defects in host defence (43–45) and the mucociliary system of the mucosa lining the middle ear cavity (46–48). The identification of a number of mouse mutants with altered ET morphology and angle have also revealed clearance as a contributing factor to the development of OME. These include the non-syndromic *Eya-4* knockout (49), the genotype-driven *Tail-short* mouse (50), the syndromic *Sh3pxd2b* (51), *Chd7* knockout (52) and the *Lmna* heterozygous mouse (53). In addition, mild craniofacial abnormalities as well as a narrowed ET have been suggested to contribute to OM development in heterozygous *Jeff* mice (*Fbxo11^+/−^*) (54,55). *Fbxo11* might also be a candidate gene for human OM, because association screens in families with high OM risk have found SNPs in the human *FBXO11* gene (56), further highlighting the importance of murine models in the translation to human diseases (57). In all of these cases, the chronic OME is predominantly bilateral. Interestingly, however, studies in children where the angle and length of the ET was measured failed to find a difference between infants with and without OME (31), indicating that other factors are likely to be involved. Therefore, the question remains whether ET anomalies underlie OM susceptibility in both syndromic and non-syndromic populations and mouse models.

To understand the mechanisms behind the high susceptibility to OME in 22q11DS mouse models, we investigated the development of the middle ear and onset of OM in *Df1/+* and *Tbx1^+/−^* heterozygous mice. In contrast to the OM mouse models mentioned earlier (such as *Eya4/+* and *Jf/+*), OME in *Df1/+* mice has been shown to be frequently unilateral (38). This therefore provides an excellent opportunity for within-animal controls to tease apart why one ear might develop OME whereas the contralateral ear appears unaffected. The OM in *Df1/+* and *Tbx1^+/−^* heterozygous mice is independent of a palate defect, indicating that in patients with 22q11.2DS, the ear disease is not caused primarily by the associated palatal defects. This observation fits with findings that the incidence of chronic OM in 22q11.2DS patients is not associated with the type of palatal abnormality (12).

*Tbx1* is expressed during development in several tissues later associated with the middle ear. From E8.0 onwards, *Tbx1* is expressed in the endodermal lining of the first pharyngeal pouch, which gives rise to the ET and parts of the epithelium lining the MEC (58–60). In addition, *Tbx1* is expressed in the mesodermal core of the arches, which give rise to the muscles of the face (61). Complete loss of *Tbx1* in homozygous mutant mice (*Tbx1^−/−^*) leads to a hypoplastic pharynx, missing ET and malformed or missing ear structures (40,59,62). The heterozygous animals, however, have been reported as being phenotypically normal with regards to these tissues (63).

In this paper, we investigate the development of the middle ear and ET in *Df1*/+ and *Tbx1^+/−^* mice, focusing on the tissues that express *Tbx1* at earlier stages of development. From our analysis, we have identified key defects in the developing tissues of the mouse mutants and have isolated the cause of the unilateral incidence of OME. Importantly, we highlight a cause of susceptibility to chronic OM not previously identified in mouse models, which will provide crucial information to aid the treatment of 22q11.2DS patients with chronic ear disease.

## Results

### Otitis media in *Tbx1^+/−^* mice resembles that observed in *Df1/+* mice

**Figure 1.**
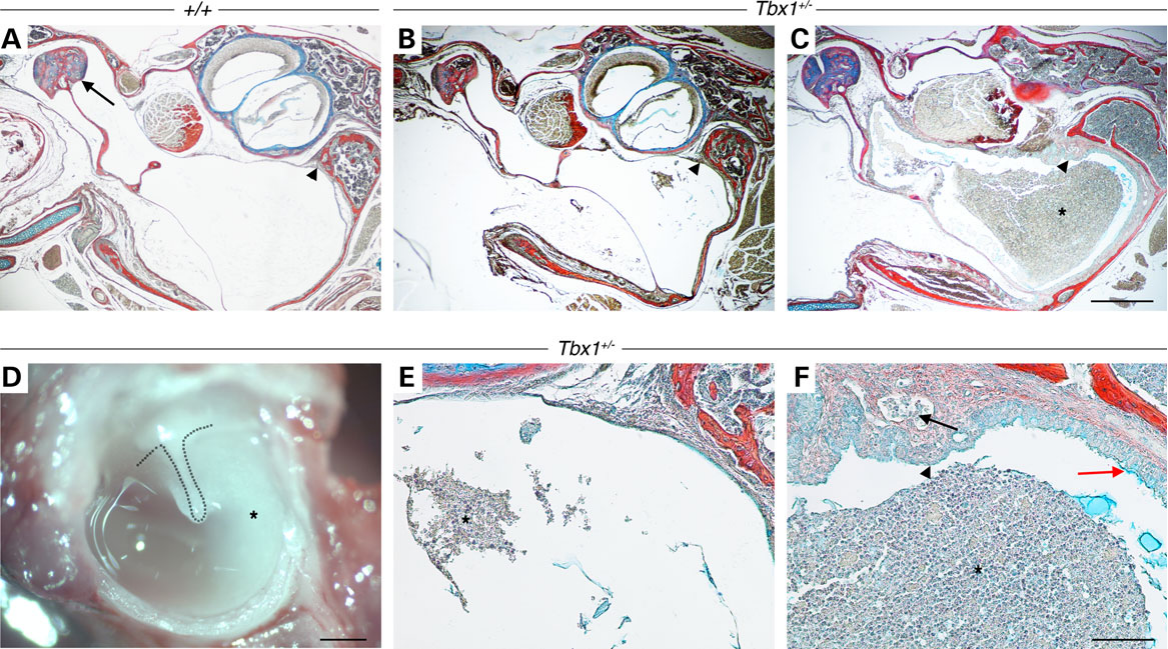
High incidence of OM in *Tbx1^+/−^* mice. (**A**) Wild-type (*+/+*), (**B**–**F**) *Tbx1^+/−^*. (A–C, E and F) Frontal trichrome-stained sections of middle ear cavities and (D) freshly dissected whole-mount preparations of auditory bullae showing the intact tympanic membrane and manubrium of the malleus (dotted line). (A) At P18, the malleus (arrow) is freely suspended in an air-filled middle ear cavity. A thin mucosa is lining the middle ear in both WT (A, arrowhead) and P18 *Tbx1^+/−^* littermate (B, arrowhead); however, *Tbx1^+/−^* mice frequently displayed patches of effusion with infiltrated cells (E, asterisk). (C) At P26, *Tbx1^+/−^* mice show a further advanced inflammation with effusion with infiltrated cells (F, asterisk), capillary hyperplasia (F, arrow), thickening of the mucosal epithelium and subepithelial connective tissue (C and F, arrowhead) as well as an increase in mucus-producing cells (F, red arrow). (D) Cloudy effusion with infiltrated cells is clearly visible in dissected auditory bullae (asterisk) in a 44-weeks-old *Tbx1^+/−^* mouse. Dorsal is top. Scale bars: A–D, 500 µm; E and F, 100 µm.

As previously described, *Tbx1^+/−^* mice develop OME (41). Histological analysis (Fig. 1A–C, E and F) and fresh whole-mount preparations of *Tbx1^+/−^* auditory bullae (Fig. 1D) revealed that 77% (10 of 13) of mice [aged postnatal day 18 (P18) to 44 weeks] displayed OM. The degree of the inflammation and quantity of effusion with infiltrated cells in these mice was very similar to that previously reported in *Df1/+* mice (38). The inflammation was observed to be either uni- (5 of 10) or bilateral (5 of 10), with no left or right prevalence in unilateral cases.

OM was found in *Tbx1^+/−^* mice as early as P18 (4 of 6), with spots of effusion and few infiltrated cells being the first sign of the disease (Fig. 1B and E). The epithelium in P18 *Tbx1^+/−^* mice at the onset of OM was thin and almost indistinguishable from the mucosa of wild-type (WT, *+/+*) littermates (Fig. 1, arrowheads in A and B). In contrast at P26, the *Tbx1^+/−^* mice showed more severe degrees of OM (Fig. 1C) categorized by proliferative changes in the mucosal lining of the middle ear cavity, effusion with infiltrated cells, increase in mucus-producing goblet cells and capillary hyperplasia (Fig. 1F). The morphological changes of the middle ear epithelium in older mice were therefore secondary to the inflammation rather than a primary cause.

### Normal Eustachian tube morphology and angles in *Df1/+* mice

**Figure 2.**
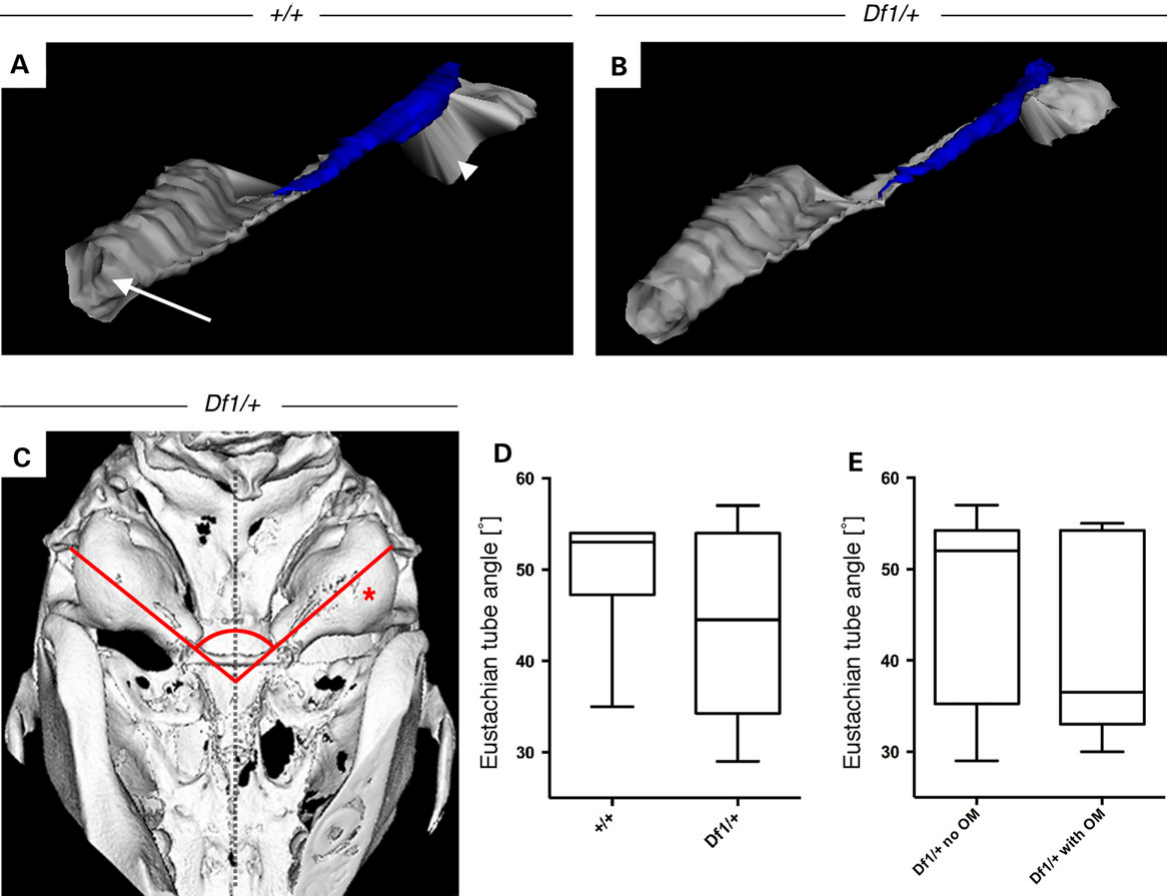
ET angles and morphology in *Df1/+* mice and WT littermates. (**A**) 3D-reconstruction of the ET (grey) from the nasopharynx (arrow) to the middle ear cavity (arrowhead) and its associated cartilage (blue) of a 11.5-week-old WT mouse and (**B**) mutant *Df1/+* littermate with OM showing no morphological differences. (**C**) Ventral view of a 3D-reconstructed microCT scan of a *Df1/+* adult mutant skull with unilateral OM on the right hand side (asterisk), showing no obvious alterations of the ET angles (red lines) from the middle ear cavity orifice to the midline of the skull (dashed vertical line). (**D** and **E**) Measurements of ET angles from 3D-reconstructed skulls of animals between the ages of 3.5 and 18.5 weeks. (D) No significant difference in ET angles comparing *Df1/+* mice (*n* = 16) with control littermates (*n* = 10) and (E) *Df1/+* mice with clear middle ear cavities (*n* = 10) to *Df1/+* mice with inflamed ears (*n* = 6). Statistical analysis was performed using Mann–Whitney test, *P* = 0.4546 and *P* = 0.5797, respectively.

As anatomical defects of the ET are the most common factors associated with OM development in human and murine disease models, we performed 3D reconstructions of the ET from histological sections. The reconstructed ET with its associated cartilage was analysed from the nasopharynx to its incision point into the middle ear cavity (Fig. 2A and B). A set of adult littermates (five *Df1/+* and one WT) was used to analyse differences in size, shape and localization of the tube. Out of these animals, four *Df1/+* mice presented with unilateral OM and varying degrees of severity of the inflammation, making them within-animal controls for the experiment in addition to a WT littermate control. Comparing the anatomy, shape and middle ear cavity orifice of the ET within *Df1/+* mice with unilateral OM as well as to a WT control, we did not observe any gross abnormalities.

In addition, measurements of ET angles were performed using micro-computerized tomography (microCT) scans of skulls from mice between the ages of 3.5 and 18.5 weeks (WT = 5, *Df1/+* = 8). The midline of the skull was used as a reference point to measure the angles to the bony orifice of the ET that connects to the MEC (Fig. 2C), resulting in two angle measurements per animal. Comparing WT angles with those of *Df1/+* mice (Fig. 2D), no significant changes could be found. Using heterozygous *Df1/+* mice as within-animal controls, we also compared ET angles of ears without the inflammation (*n* = 10) to mutants exhibiting OM (*n* = 6; Fig. 2E); however, this analysis revealed no significant differences.

MicroCT scans were also used to determine morphological changes of the auditory bulla, the neural crest-derived bony structure surrounding the middle ear cavity, as reduced auditory bullae volume has previously been linked to impact on cavitation of the middle ear (64). Measuring both length and width of the hollow capsule, we found a significant reduction in auditory bulla size (Supplementary Material, Fig. S1). However, smaller bulla size did not correlate with incidence of inflammation and no retained mesenchyme was observed in P18 *Tbx1^+/−^* mice with effusion (Fig. 1B and C), indicating that other factors may cause the high susceptibility to OM.

### *Df1/+* ears without OM display normal cilia density and distribution

**Figure 3.**
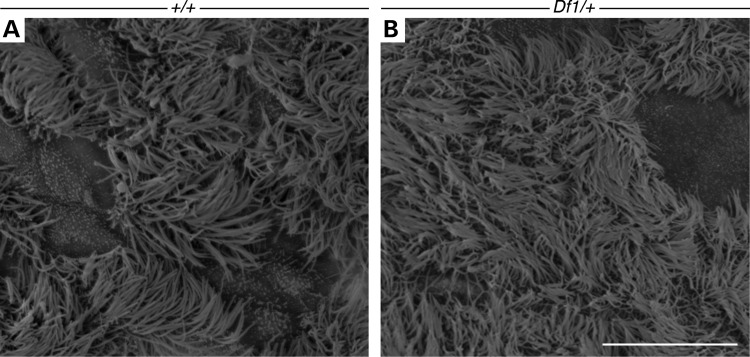
*Df1/+* ears without OM display normal cilia density and distribution. (**A**) 17-week-old WT mouse. The epithelium adjacent to the ET and cochlea is highly ciliated with patches of unciliated epithelium. (**B**) 38-week-old *Df1/+* mutant without OM. The epithelium shows a similar ciliary integrity despite the old age of this mouse. Scale bar: 10 µm.

As OM is often associated with impaired mucociliary clearance, we examined the ciliary integrity in *Df1/+* mice. Scanning electron microscopy (SEM) have previously revealed that *Df1/+* mice with OM have reduced cilia density, with the cilia rarefied and shortened (38). We therefore wanted to investigate the cilia in mutant ears without any signs of OM to see whether the cilia could be a causative factor. In WT and *Df1/+* mice without OM, the mucosa between the ET and cochlea displayed a thick lawn of evenly distributed cilia (Fig. 3A and B). No difference was observed between the cilia shape and distribution, indicating that changes in ciliary integrity are secondary to the inflammation, as described in other OM mouse models (65).

### Adult *Tbx1^+/−^* mice demonstrate an impaired clearing function of the Eustachian tube

**Figure 4.**
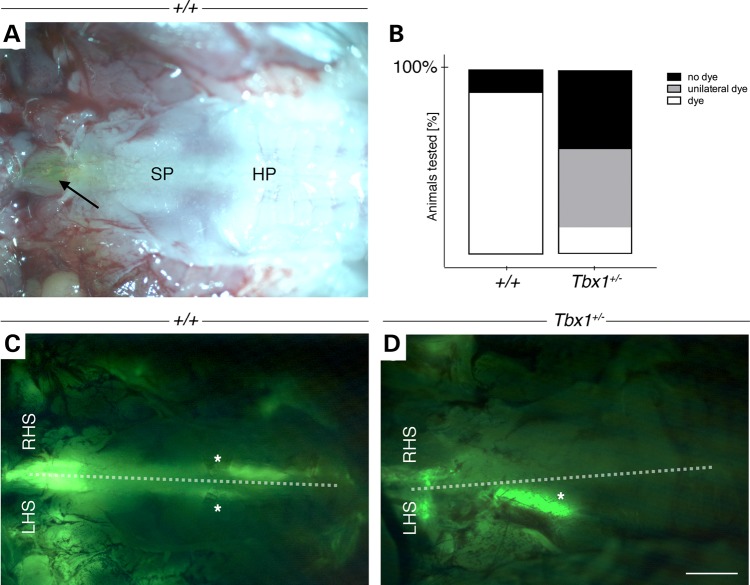
Functional test of ET patency in adult *Tbx1^+/−^* mice. (**A** and **C**) WT (+/+); (**D**) *Tbx1^+/−^*. (A) Ventral view of the soft palate (SP) and hard palate (HP) with fluorescent dye visible in the nasopharynx to pharynx opening (arrow). Right is frontal. (C) Same WT animal as (A) with fluorescent dye (indicated by the asterisk) visible on both left hand side (LHS) and right hand side (RHS) of the palatal midline (dashed white line). (D) In *Tbx1^+/−^* mice, the dye was frequently observed only on one side, here the LHS of the palate (asterisk). (**B**) Column graph displaying the percentage of animals with different dye clearing efficiency of the ET, classified as no dye in pharynx (black), dye only on one side of the soft palate (grey) and dye in the pharynx (white). Analysed were 3 litters between the ages of P18 and P21. Five minutes after the dye injection, we observed 88% (14 of 16) of WT mice with and 12% (2 of 16) without dye in the pharynx region. In contrast, the minority of *Tbx1^+/−^* mice (14%, 1 of 7) showed dye in the pharynx. 43% (3 of 7) either presented with no dye or unilateral dye on the right (1 of 3) or left (2 of 3) hand side of the palate. Scale bar: A, C, D 1 mm.

Even though no morphological defects of the ET were evident from the 3D-reconstructions, and cilia integrity appeared normal, the question remained whether the ET of *Tbx1^+/−^* mice was fully functional. We therefore performed a functional analysis of ET patency to determine whether the ET in *Tbx1^+/−^* mice could clear dye from the middle ear cavity through to the nasopharynx and into the oral cavity. For the analysis, we chose juvenile mice between the ages of P18 and P21 as the middle ear was completely cavitated, yet the OM had only just initiated (see Fig. 1B and E). A trial was first carried out using P18 WT mice. A fluorescent dye was injected into the middle ear under terminal anaesthesia, and the time that the dye took to appear in the nasopharynx assessed. Five minutes after injection of the fluorescent dye, it was visible through both soft and hard palate giving the opportunity to trace whether the dye was cleared from both or only one ear (Fig. 4A and C)(*n* = 4). When WT mice were culled 2–3 min after injection of the dye, no dye appeared in the palate or nasopharynx/pharynx entrance (*n* = 2). Five minutes was therefore selected as the time that WT animals were able to clear dye through their ETs. In total, we analysed three litters consisting of 16 WT and 7 *Tbx1^+/−^* mice. Mice included in the study were within a set weight range (between 4.7 and 8.8 g), and no significant difference in weight was observed between WT and *Tbx1^+/−^* mice or between male and female mice (one-way ANOVA, *P* = 0.0954). In 88% of WT animals, the dye was cleared from the cavity and appeared in the palate and pharynx region 5 min after the injection (Fig. 4B). In contrast, after the same time interval, the dye was found in the pharyngeal region in only 14% of *Tbx1^+/−^* mice. In the other *Tbx1^+/−^* mice, the dye was either not cleared at all (43%) or only visible on one side of the palate (43%; Fig. 4D), indicating impairment of and higher variability in the clearing ability of the ET in *Tbx1^+/−^* mice.

### Adult *Df1/+* mice have hypoplastic Eustachian tube muscles

**Figure 5.**
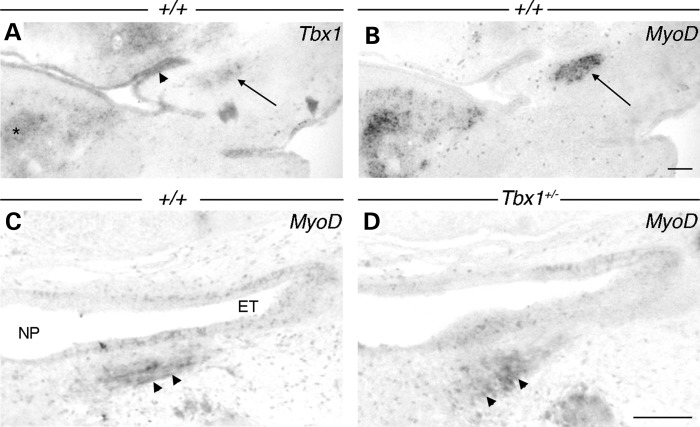
Embryonic expression of *Tbx1* and *MyoD* in ET muscles. (**A**–**C**) WT (+/+); (**D**) *Tbx1^+/−^*. (A) At E12.5, *Tbx1* is expressed in the early ET (arrowhead) and adjacent branchiomeric muscle masses (arrow). Note *Tbx1* is also expressed in forming tongue muscles (asterisk). (B) The same ET muscle masses express the MRF *MyoD* (arrow). (C) At E15.5, *MyoD* is expressed in muscle fibres of the maturing mLVP (arrowheads) next to the nasopharynx (NP) and ET, whereas in *Tbx1^+/−^*, *MyoD* appears to be diffusely expressed (arrowheads). Scale bars: 200 µm.

Having ruled out a morphological defect of the ET but yet observing an impaired ET patency, we turned to analysing ET-associated muscles. *Tbx1* is expressed in the mesodermal core of the pharyngeal arches during development (66,67). At E12.5, *Tbx1* is expressed in the mesoderm that gives rise to the muscles of the ET (Fig. 5A). This muscle mass includes the first pharyngeal arch-derived medial pterygoid and tensor veli palatini muscle (mTVP) as well as the fourth pharyngeal arch-derived levator veli palatini muscle (mLVP) (68–71). *Tbx1* has been shown to regulate the onset of branchiomeric myogenesis by activation of myogenic regulation factors (MRFs), with *MyoD* expression overlapping with *Tbx1* (Fig. 5A and B). Muscle masses derived from the first and second pharyngeal arches have been shown to be missing in *Tbx1^−/−^* mice (72), whereas the fourth pharyngeal arch was absent (40). The muscles in the *Tbx1^+/−^* mice, however, have been reported to be normal (72).

**Figure 6.**
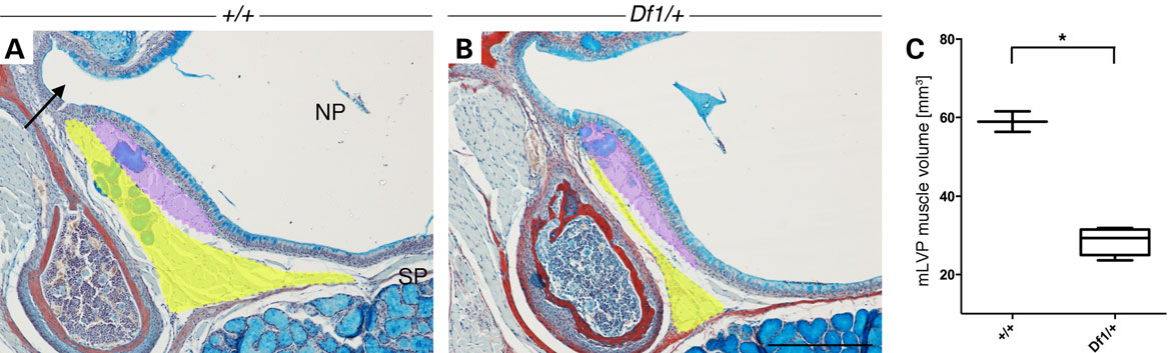
Muscle involved in ET opening is hypoplastic in adult *Df1/+* mice. (**A** and **B**) Trichrome-stained frontal sections of the (A) nasopharynx (NP) and frontal portion of the ET (arrow) showing the levator veli palatini (mLVP, yellow) and Palatopharyngeus muscle (mPP, purple) inserting into the soft palate (SP) in 11.5-week-old control littermates. (B) *Df1/+* mice show a reduction in muscle size. (**C**) Measurements of the mLVP (yellow) volume from histological frontal sections show that muscles in adult *Df1/+* animals (*n* = 8) are significantly smaller compared with those of control littermates (*n* = 2; *P* < 0.0444). Statistical analysis was performed using non-parametric Mann–Whitney test. Scale bars: A and B, 200 µm.

We examined the mLVP in adult mice heterozygous for the multigene deletion (*Df1/+*) and *Tbx1* (*Tbx1^+/−^*). Histological analysis of serial sections from the nasopharynx to the middle ear cavity showed that the mLVP was present in both WT and *Df1/+* mice (Fig. 6A and B, respectively, yellow), running inferior to the ET along its distal axis (Fig. 6A arrow). No differences were found in either the muscle insertion point into the midline of the soft palate or the general fascicle direction; however, the mLVP appeared smaller in size. To confirm this observation, the whole mLVP was analysed using ImageJ and PRISM GraphPad to calculate the muscle volume, which revealed a significantly smaller muscle size in adult *Df1/+* mice compared with their WT littermates (Fig. 6C).

### Early onset of muscle defect in both *Df1/+* and *Tbx1^+/−^* neonatal mice

**Figure 7.**
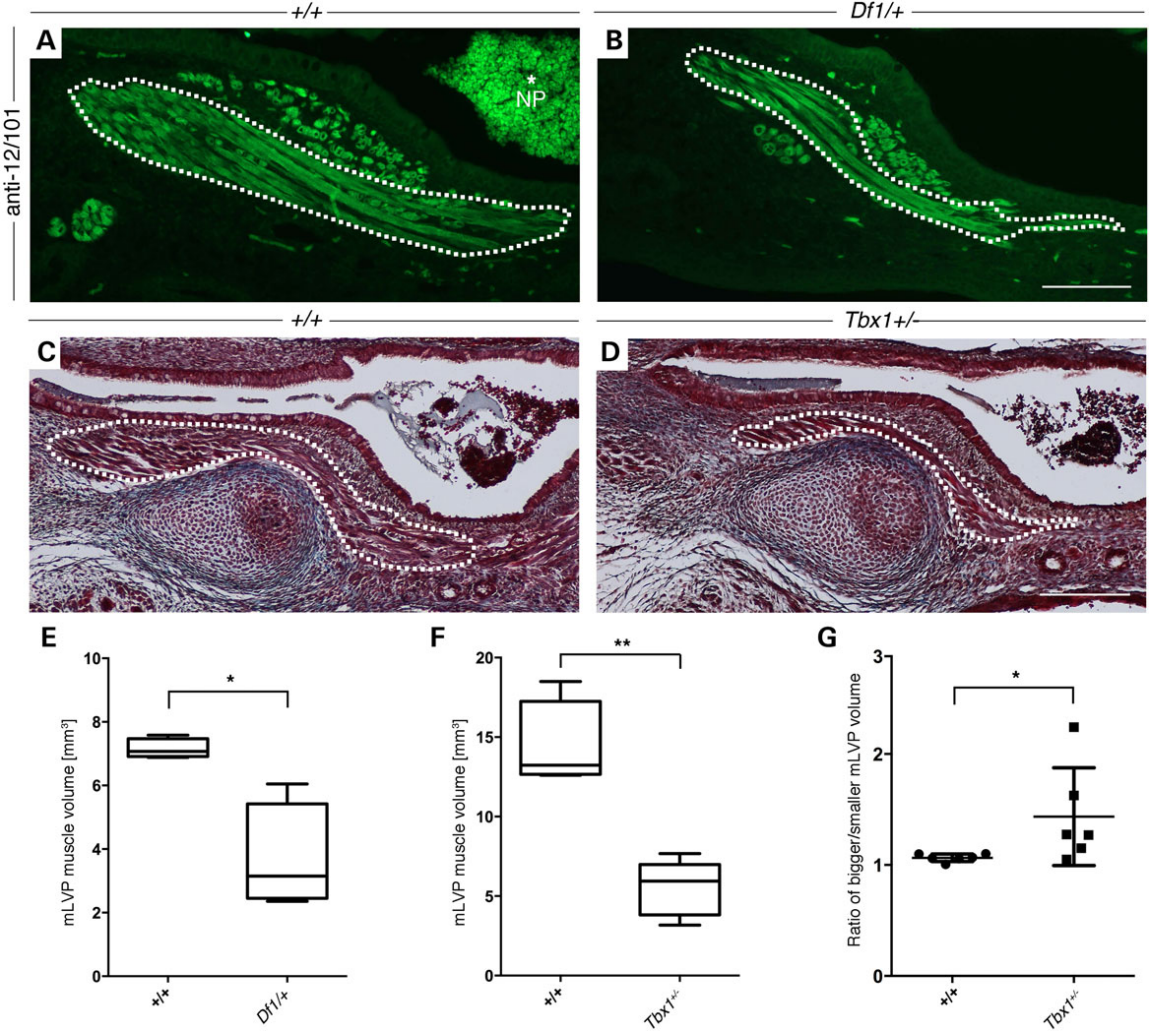
Neonatal *Df1/+* and *Tbx1^+/−^* mice have hypoplastic mLVP. (**A** and **C**) WT; (**B**) *Df1/+*; (**D**) *Tbx1^+/−^*. The mLVP is outlined in white. (A and B) Immunohistochemistry against the skeletal muscle marker 12/101 stains the muscle fascicles of the mLVP in newborn control mice (A) and in *Df1/+* littermates, showing a reduced muscle fascicle number. (D) A reduced muscle size can also be found in Masson's Trichrome-stained *Tbx1^+/−^* mice compared with (C) control littermates. (**E** and **F**) Measurements of the mLVP show that muscle masses in neonatal *Df1/+* (*n* = 4, E) and *Tbx1^+/−^* (*n* = 6, F) animals are significantly smaller compared with their control littermates (WT = 4 and WT = 6, respectively; *P* = 0.0286 and 0.0022, respectively). (**G**) Scatter plot graph displaying the ratio of the two mLVP within each animal, demonstrating a significant size difference between these muscles in neonatal *Tbx1^+/−^* mice (WT = 6, *Tbx1^+/−^* = 6; *P* = 0.0411). Statistical analysis was performed using non-parametric Mann–Whitney test. Neonatal ages were ranging between E18.5 and P1. Note accumulated blood in the nasopharynx (NP; A, asterisk) occurred post-mortem. Scale bars: A and B, 100 µm; C and D, 200 µm.

The observation that adult *Df1/+* mice have smaller ET-associated muscles raises the question of whether hypoplastic muscles are secondary to the chronic inflammation of the middle ear or caused by a myogenic defect in early development that reduces muscle size before the onset of the disease in the uncavitated middle ear. We therefore performed immunostaining using an antibody against the skeletal muscle marker 12/101 in newborn mice, which labelled individual muscle fascicles of the mLVP inferior to the ET and next to the nasopharynx. The number of fascicles and the general size of the muscle appeared reduced in *Df1/+* mice compared with WT littermates (Fig. 7B and A, respectively). Muscle measurements were performed as mentioned before using histologically stained frontal serial sections, revealing a significant reduction in volume of the mLVP in neonatal *Df1/+* mice (Fig. 7E). The same significantly smaller volume of the mLVP was also found in P1 *Tbx1^+/−^* mice using Masson's Trichrome-stained histological sections (Fig. 7C, D and F). To investigate the onset of the defect, we compared myogenesis in *Tbx1^+/−^* and WT embryos at E15.5. This is the first time point when the mLVP can be distinguished from the other neighbouring muscles, with the muscle precursors at earlier stages forming a combined mass (Fig. 5B and C). *MyoD* staining showed a subtle difference in the developing mLVP at this time point, with the WT muscle appearing as a thin band of fibres whereas the *Tbx1^+/−^* mLVP was more diffuse without an obvious orientation (Fig. 5C and D). These results indicate that *Tbx1* is the primary gene responsible for the myogenic defect, which is evident as soon as the mLVP can be detected.

Interestingly, when assessing the mLVP volume on either side of the head within the same animal, we frequently found a significant difference in severity of the defect in the *Tbx1^+/−^* mice. In WT mice, when the muscle volume on one side was divided by the other side, the ratio equalled 1, indicating symmetry. However, in the *Tbx1^+/−^* mice, when the ratio was compared (bigger muscle divided by smaller muscle), a clear asymmetry within the same animal was observed (Fig. 7G).

### Asymmetry and smaller muscle size in juvenile *Tbx1^+/−^* mice correlates with the development of Otitis media

**Figure 8.**
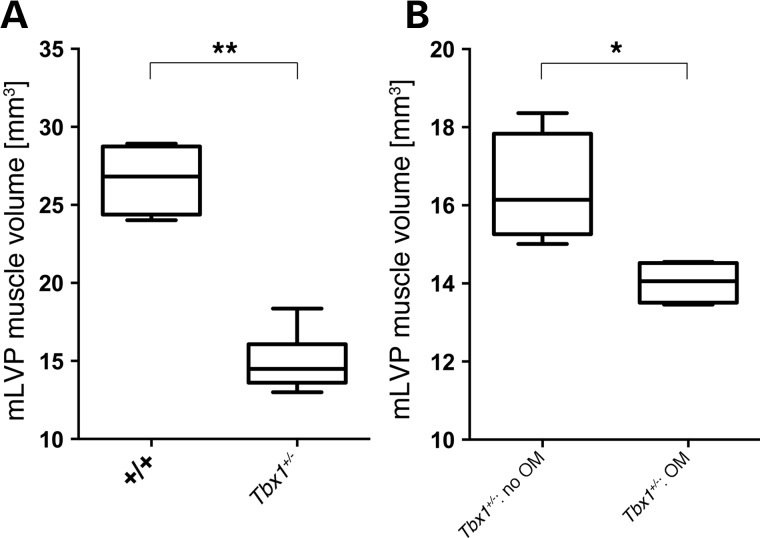
mLVP volume is significantly reduced in inflamed ears of juvenile *Tbx1^+/−^* mice. (**A**) Comparison of mLVP volume in WT (*n* = 4) and *Tbx1^+/−^* (*n* = 10) littermates showed a significant reduction of muscle size in mutant mice (*P* = 0.002). (**B**) Boxplot graph displaying correlation between muscle volume and early signs of the inflammation within *Tbx1^+/−^* animals. Muscles in inflamed ears (*n* = 4) were significantly smaller than the muscles in ears without signs of an inflammation (*n* = 4; *P* = 0.0286). Statistical analysis was performed using non-parametric Mann–Whitney test. One animal was excluded from the analysis because of infiltrated blood cells that occurred post-mortem.

The variation in mLVP volume across and within the *Tbx1^+/−^* and *Df1/+* population could explain the observed variation in susceptibility to OM. We therefore assessed the muscle volume in *Tbx1^+/−^* mice and correlated the muscle size to the presence of uni- or bilateral OM. The onset of OM, as shown by patches of effusion with a few infiltrated cells, was observed as early as P18 (Fig. 1B and E), and so this stage was chosen for the analysis. As expected, comparing the overall muscle size of the mLVP in WT with *Tbx1^+/−^* littermates, we found a significant reduction in the mLVP of *Tbx1^+/−^* mice (Fig. 8A). To assess whether the muscles in the vicinity of the ET were hypoplastic, we also examined the first arch-derived tensor tympani muscle (mTT), which originates from the anterior tubal cartilage and runs posterior-laterally along the ET before entering the middle ear cavity. Interestingly, this muscle was also significantly reduced in size, however, to a lesser degree then the mLVP in the same animals, indicating not all muscles are affected to the same extent (Supplementary Material, Fig. S2 and Table S1).

We then correlated the mLVP volume to inflamed and uninflamed ears in the mutant mouse population with either uni- or bilateral OM and found that the mLVP was significantly smaller in inflamed ears compared with ears without OM, demonstrating a direct correlation between mLVP muscle size and development of the disease (Fig. 8B; Supplementary Material, Table S1). Importantly, a similar correlation was not observed when the neighbouring, non-ET muscle (the mTT) was assessed. Therefore, the link between ear disease and muscle volume appears to be only valid for the ET muscles (Supplementary Material, Fig. S2 and Table S1).

## Discussion

Here, we present a novel mechanism by which reduced *Tbx1* expression leads to either uni- or bilateral incidence of OM. Using mouse models, we show that changes to the size of a key ET muscle, the levator veli palatini (73), during development impact on the ability of the ET to clear debris and secretions from the middle ear after birth, resulting in increased susceptibility to developing OME.

ET function is regulated by two paratubal muscles, which contract in a sequential manner, thereby allowing a discrete air bolus to travel through the ET into the MEC (68). The specific contributions of the paratubal muscles are controversial (69,74). Recent advances suggest that the first arch-derived tensor veli palatini muscle opens the posterior portion of the ET, whereas the fourth pharyngeal arch-derived levator veli palatini muscle functions in opening and closing of the anterior pharyngeal portion (68,75). Myogenic defects in the mLVP have been implicated in tubal dysfunction in humans and are likely to increase the risk of OM development (73).

*Tbx1* is expressed in the mesodermal core of the pharyngeal arches from as early as E9.5 (59). Previous studies have shown that head muscles, derived from pharyngeal arch mesoderm, are hypoplastic or missing when *Tbx1* is homozygously deleted. The phenotype has been attributed to increased apoptosis, which reduces the muscle precursor pool in the mesodermal core, and an impaired activation of MRF genes during myogenesis. In heterozygous mice, however, analysis of the first and second pharyngeal arch muscles showed no alteration in muscle size (41,72), indicating that these muscles are able to develop correctly with half the normal dose of *Tbx1*.

In this study, we concentrated on intrinsic ET muscles derived from caudal arches, instead of cranial arches. We performed volumetric measurements of the fourth arch-derived levator veli palatini muscle in *Tbx1^+/−^* and *Df1/+* mice. Volumetric analysis is a much more sensitive method to gauge size compared with the use of histological sections or whole-mount preparations. Hypoplastic muscles were observed in 100% of mutants examined, ranging from adult 11.5-week-old mice presenting with severe inflammations to E18.5 embryos with uncavitated ears, indicating that a reduction of muscle size was not a secondary consequence of the disease. A defect in the expression domain of *MyoD* was also apparent at E15.5, when the mLVP is first identifiable as a distinct muscle. Subtle changes in the expression of *MyoD* therefore appear to underlie the eventual smaller size of this muscle.

The most caudal and ventral structures in the developing embryo show the strongest expression of *Tbx1* (61,62,76). *Tbx1* also functions during pharyngeal segmentation, which has been established by timed mutation and cell-fate mapping experiments and is expressed in a rostro-caudal gradient following the direction of pharyngeal segmentation (59,77). As such, ablation of *Tbx1* results in an unsegmented pharynx and the absence of PP2–4 and PA3–6, with PA2 being hypoplastic and PA1 present but abnormally patterned (40,72). Taken together, more caudal arches (PA3–PA6) seem to be more severely affected than cranial regions. The observation is underlined by other phenotypes being more penetrant in caudal arches, such as neural crest cell abnormalities. *Tbx1* expressed in the surface ectoderm acts upstream of the homeobox gene Gastrulation brain homeobox (*Gbx2*), which is required for normal pharyngeal arch artery development by providing directional cues to cardiac NCCs to migrate into caudal arches (78). In the absence of *Gbx2*, NCC migration into PA3, 4 and 6 is altered, leading to defective 4PAA development, a hallmark feature of 22q11.2DS patients (78). Cranial nerves are partly derived from NCCs and display abnormal migratory pathways and fusion in *Tbx1^−/−^* mice, including the most rostral (trigeminal and facial) and more caudal (glossopharyngeal and vagus) nerves (59). In mice heterozygous for *Tbx1*, ganglia fusion has only been observed for the most caudal nerves, indicating that caudal arches are more sensitive to altered *Tbx1* dose than rostral pharyngeal structures (79). Other temporal-spatial signals have been implicated in pharyngeal patterning such as the retinoic acid (RA) signalling pathway. Even subtle disruptions of this pathway by either decreased or increased expression of genes involved in RA metabolism have been shown to be detrimental to caudal arch development and result in 22q11.2DS-like defects (80). *Tbx1* has been linked to the RA pathway because RA-degrading enzymes, such as CYP26A1, have been shown to be downregulated in microarray studies of *Df1/+;Tbx1^+/−^* transheterozygotes (81). Interestingly, expression studies also indicate that *Cyp26a1* is reduced in *Tbx1^+/−^* mice (80), suggesting that in mice with half the dose of *Tbx1*, the development of the caudal pharyngeal apparatus is also particularly sensitive to altered *Tbx1* dose. Our analysis of muscle volume also indicates that rostral muscles are less affected compared with more caudally derived muscles, with a much smaller mLVP (fourth arch) compared with the mTT (first arch), agreeing with findings that half the dose of *Tbx1* is more likely to cause defects in tissues and organs derived from caudal arches rather than rostral arches (40,66,79). However, we did observe a significant reduction in size of the mTT, indicating that all cranial muscles are influenced by *Tbx1* dosage.

Although all heterozygous mutants had hypoplastic muscles, the degree of hypoplasia differed not only between littermates but also between ears within the same animal (contralateral sides) and between different muscles. The variability in mLVP muscle volume was directly correlated with unilateral OM, showing that the side presenting with inflammation was associated with significantly smaller ET muscles than the contralateral unaffected ear. The results were significant despite using only a small data set. The same correlation was not found for the mTT. This muscle is attached to the manubrium and protects the ear against loud noises, and despite running along the ET has no functional implication in ET opening and closing. Although reduced in size, the mTT was not significantly smaller in inflamed ears compared with uninflamed ears (Supplementary Material, Fig. S2). This data indicate that high susceptibility to ear disease is linked to defects in ET muscles specifically, rather than owing to a general muscle hypotonia.

In *Tbx1^−/−^* mice, sporadic and hypoplastic muscles display normal patterning, proliferation and differentiation properties once determined, indicating that *Tbx1* is not absolutely required for MRF activation but initiates their expression in a robust and bilateral manner (72). The observed asymmetry in both homozygous and heterozygous mutants might therefore underlie a stochastic mechanism in premyoblast specification.

Interestingly, we found that some of the *Tbx1* heterozygotes were able to successfully clear dye from the middle ear into the pharynx on one side of the head but not the other. Such unilateral clearance was not observed in any of the WT mice we tested. The asymmetry in muscle volume between both ET muscles within *Tbx1^+/−^* mice underlines the dosage-dependent role of *Tbx1* in the robust and bilateral activation of MRF genes during myogenesis of pharyngeal arch-derived muscles (72).

Whether the condition of the hypoplastic levator veli palatini muscle is responsible for an insufficient clearance of the ET owing to weaker contraction and strength or whether the muscle produces defects in the elevation of the soft palate which then obstructs the opening and closing of the tube remains unclear. However, the ET functional tests imply a direct influence of the muscle on ET patency.

Other common causes of the inflammation such as defects in the mucociliary clearing apparatus contributing to the development of OM cannot be excluded; however, SEM of adult *Df1/+* mice without OM showed normal cilia morphology and distribution. In addition, 22q11.2DS mouse models are not reported to have a cilia defects and do not display any phenotypes normally associated with immotile cilia such as *situs inversus* or infertility, indicating that half the dose of *Tbx1* is sufficient for normal cilia function.

This is the first OM mouse model to link a myogenic defect to the development of inflammation. Previous models have implicated defects in the angle or morphology of the ET itself (49,52,53,55). Our findings suggest new strategies for treatment and particularly prevention of the inflammation in susceptible individuals. Currently, OM is mainly treated by the insertion of ventilation tubes into the tympanic membrane to help drain effusions from the middle ear cavity. This is not only the most common operation in the UK (82) but also entails complications such as tympanic membrane perforation, atrophy and the development of cholesteatomas (83).

As the size of the ET muscles appears to be a crucial factor in OM development in both *Tbx1^+/−^* and *Df1/+* mice, we hypothesize that in 22q11.2DS patients targeted training of specific muscle masses associated with the ET and soft palate might reduce susceptibility to the disease. We therefore suggest more trials on alternative treatments, such as ET rehabilitation therapy. This is a controversial training method for lessening symptoms of ET dysfunction by exercising paratubal muscles (84) and might serve as an alternative non-invasive therapy for 22q11.2DS patients. Currently, there is only limited data available on trials regarding the specific training of head and neck muscle groups. In the case of decreased respiratory abilities, however, exercise of ventilatory muscles has proven a successful therapy, resulting in a rapid improvement of muscle strength and swallowing performance (85). Alternatively, auto-inflation of the ET may present a possible method for treating OM.

We have shown that subtle developmental defects in the expression of *MyoD* and later the size of muscle populations derived from the fourth pharyngeal arch can lead to later susceptibility to OM. Although our work here focuses on the role of *Tbx1*, other genes with roles in early muscle initiation and specification may also lead to similar increased susceptibility. For example, *Eya1* and its cofactor *Six1* are downregulated in caudal arches of *Tbx1^−/−^* mice and recapitulate most 22q11.2DS features when homozygously deleted (86). *Eya1* is expressed in the mesodermal core during early muscle development and in adult skeletal muscles (87). In addition, patients with mutations in *EYA1* have various ear defects including high susceptibility to OM (88,89). *Six1^−/−^; Eya1^−/−^* mice have severely hypoplastic branchiomeric muscles (86), suggesting an additional role of these genes in branchiomeric myogenesis. Ectopic expression of the transcriptional complex has been shown to reprogram adult skeletal muscles by inducing a fibre type transition from the slow (type I) to the fast type (type II) (87). Zim *et al*. (90) examined another fourth arch-derived muscle, the pharyngeal constrictor muscle in human 22q11.2DS patients, revealing an increase in fibre type I, suggesting that Tbx1 might impact on muscle fibre type. However, no changes in the fibre composition were found in the masseter and pharyngeal constrictor in *Tbx1^+/−^* mice (91). As the levator veli palatini muscle and tensor veli palatini muscle are predominantly composed of fibre type I, a muscle fibre-related contribution to muscle hypotonia in mutant mice appears unlikely, suggesting that the main cause for the impaired clearing function in *Tbx1^+/−^* and *Df1/+* mice is muscle hypoplasia owing to smaller muscle size. This is also highlighted by the observation that the caudal arch-derived pharyngeal constrictor muscle was shown to be significantly thinner in 22q11.2DS patients (90), indicating that muscle masses derived from caudal arches are generally smaller.

## Material and Methods

### Animals

The generation of *Df1/+* and *Tbx1^+/−^* mutant mice has been described (37,66). In brief, both lines had been maintained on a C57BL/6 background for a minimum of 10 generations prior to the analyses. The deletion itself was engineered on a 129S5 SvEvBrd genetic background. Mice were housed in groups in Home Office-approved conditions, which did not increase susceptibility to infections in control mice.

### Middle ear histology and staining

Mouse heads were fixed overnight in 4% paraformaldehyde (PFA) at 4°C and then, depending on the age, decalcified in EDTA Solution [67.5% EDTA, 7.5% PBS and 25% PFA (4%)]. The tissue was dehydrated through a methanol series and isopropanol and cleared in tetrahydronaphthalene before embedding in paraffin wax. The 9-μm frontal sections were mounted on Superfrost Plus Slides, dewaxed in Histoclear and rehydrated through IMS.

For Trichrome staining, the slides were stained with 1% Alcian blue in 3% acetic acid pH 2.5, Ehrlich's haematoxylin and 0.5% Sirius Red in saturated picric acid.

For muscle measurements, Masson's Trichrome staining was used to distinguish muscle (red) and cells from surrounding connective tissue and collagen fibres (blue). The slides were incubated in Weigert's Haematoxylin, stained in running water and Masson's Stain, differentiated in 5% Phosphomolybdic/5% Phosphotungstic Acid and immersed in 1% acetic acid. For both staining methods, slides were then dehydrated, incubated in Histoclear and mounted in Neo-Mount. Slides were viewed and photographed under bright field using a Nikon Digital Sight Camera.

For whole-mount dissections, mice were culled by cervical dislocation and the auditory bulla was carefully dissected by removing the outer ear, leaving the tympanic membrane intact. Pictures were taken with a dissection microscope (Leica MZFiii) and Leica DFC300 camera.

### Scanning electron microscopy

The middle ears of adult mice were dissected and the middle ear mucosa exposed by removing the outer ear, eardrum, tympanic ring and the malleus and incus. Ears were then fixed in 2.5% glutaraldehyde in 0.15 m cacodylate buffer (pH 7.2) overnight at 4°C and washed and post-fixed in 1% osmium tetroxide. Specimens were then dehydrated through an ethanol series and dried using a Polaron E3000 critical point dryer. After mounting and coating with gold (Emitech K550X sputter coater), the surface of the mucosa was examined and images recorded using a Hitachi S-3500N scanning electron microscope operating at 10 kV in high vacuum mode.

### Immunohistochemistry

Slides were deparaffinized, rehydrated to PBS and incubated in Tris–EDTA Buffer, pH 9 for 30 min at 95°C for heat-induced antigen retrieval. Sections were permeabilized in 1% Triton X-100 in PBS and blocked in Blocking Solution (10% goat serum and 1% BSA in PBT) for 1 h at room temperature before incubation in the primary antibody 12/101 (DSHB, mouse, 1 : 100) overnight at 4°C. The secondary antibody Alexa Fluor 488 anti-mouse (Invitrogen) was diluted 1 : 500 in Blocking Solution and incubated for 2 h at room temperature. After washing in PBS, the slides were mounted using Vectashield mounting medium. Slides were photographed with an Axioscop 2 plus microscope (Axio Cam HRc).

### *In-situ* hybridization on paraffin slides

Digoxigenin-labelled RNA antisense probes for *Tbx1* and *MyoD* were prepared by standard methods. The probes used for *Tbx1* (61) and *MyoD* (92) were all previously described. *In-situ* hybridization on frontal sections was performed according to previously published methods (93).

### Muscle measurements

Measurement of the levator veli palatini muscle was performed from histologically stained serial sections using Image J. Measurements taken from either every (neonatal mice) or every second slide (P18 and older mice) were interpolated using a fourth-order polynomial regression model created in PRISM GraphPad. The area under the curve represented the precise volume of the muscles and was therefore used for statistical analysis. All muscle measurements were analysed using a Student's *t*-test. A non-parametric Mann–Whitney test was also significant when applied to the obtained measurements.

### 3D reconstruction of the Eustachian tube

Serial histologically stained sections were used for 3D reconstruction. A digital picture of the ET from the nasopharynx to the middle ear cavity was taken for each ear of every other section. To contour the area of interest and to generate the 3D surface of the ET, WinSurf Software was used. Alignment of the stack was performed by eye, and measurements were performed in Image J.

### Micro-computerized tomography

MicroCT was used for the 3D analysis of *Df1/+* and WT skulls. Adult mouse heads were fixed in 4% PFA over night at 4°C. After washing in PBS, they were scanned using a GE Locus SP micro scanner (GE Pre-clinical Imaging, London, Ontario, Canada). Auditory bulla size and angle measurements of the ET were performed using MicroCT images, MicroView Program and Image J.

### Functional test of the Eustachian tube

Postnatal day 18 (P18) and P21 old mice were terminally sedated using a 1 : 2:1 solution of fentanyl citrate and fluanisone anaesthesia (Hypnorm, VetaPharma), ddH_2_O and midazolam hydrochloride (Hypnovel, Roche) at a dosage of 5 µl per 1 g. After sedation occurred, the tympanic membrane was carefully punctured and mice were injected with a 1% aqueous solution of the fluorescent tracer Fluorescein (Sigma) into both middle ear cavities and left in a prone position. Mice were culled by cervical dislocation after a validated time of 5 min. To visualize the soft palate and nasopharynx to pharynx orifice, the mandible and tongue were dissected and photographed in a supine position to allow a ventral view of the palate using a dissection microscope (Leica MZFiii) and Leica DFC300 camera with fluorescence.

### Ethics statement

All *in vivo* experiments were conducted in accordance with the United Kingdom's Animal (Scientific Procedures) Act of 1986, under a project license approved by the UK Home Office.

## Supplementary Material

Supplementary Material is available at *HMG* online.

## Funding

The work was supported by Action on Hearing Loss (S24) and Medical Research Council (G1001232). Funding to pay the Open Access publication charges for this article was provided by King's College London from their block grant from RCUK.

## Supplementary Material

Supplementary Data

## References

[1] Thomas J.A., Graham J.M. (1997). Chromosomes 22q1l deletion syndrome: an update and review for the primary pediatrician. Clin. Pediatr. (Phila).

[2] Devriendt K., Fryns J.P., Mortier G., van Thienen M.N., Keymolen K. (1998). The annual incidence of DiGeorge/velocardiofacial syndrome. J. Med. Genet..

[3] Ryan A.K., Goodship J.A., Wilson D.I., Philip N., Levy A., Seidel H., Schuffenhauer S., Oechsler H., Belohradsky B., Prieur M. (1997). Spectrum of clinical features associated with interstitial chromosome 22q11 deletions: a European collaborative study. J. Med. Genet..

[4] Puech A., Saint-Jore B., Funke B., Gilbert D.J., Sirotkin H., Copeland N.G., Jenkins N.A., Kucherlapati R., Morrow B., Skoultchi A.I. (1997). Comparative mapping of the human 22q11 chromosomal region and the orthologous region in mice reveals complex changes in gene organization. Proc. Natl Acad. Sci. USA.

[5] Rauch A., Zink S., Zweier C., Thiel C.T., Koch A., Rauch R., Lascorz J., Hüffmeier U., Weyand M., Singer H. (2005). Systematic assessment of atypical deletions reveals genotype-phenotype correlation in 22q11.2. J. Med. Genet..

[6] Jaquez M., Driscoll D.A., Li M., Emanuel B.S., Hernandez I., Jaquez F., Lembert N., Ramirez J., Matalon R. (1997). Unbalanced 15;22 translocation in a patient with manifestations of DiGeorge and velocardiofacial syndrome. Am. J. Med. Genet..

[7] Scambler P.J. (2000). The 22q11 deletion syndromes. Hum. Mol. Genet..

[8] Epstein J.A. (2001). Developing models of DiGeorge syndrome. Trends Genet..

[9] Kobrynski L.J., Sullivan K.E. (2007). Velocardiofacial syndrome, DiGeorge syndrome: the chromosome 22q11.2 deletion syndromes. Lancet.

[10] Lindsay E.A., Baldini A. (1998). Congenital heart defects and 22q11 deletions: which genes count?. Mol. Med. Today.

[11] Gerdes M., Solot C., Wang P.P., Moss E., LaRossa D., Randall P., Goldmuntz E., Clark B.J., Driscoll D.A., Jawad A. (1999). Cognitive and behavior profile of preschool children with chromosome 22q11.2 deletion. Am. J. Med. Genet..

[12] Reyes M.R., LeBlanc E.M., Bassila M.K. (1999). Hearing loss and otitis media in velo-cardio-facial syndrome. Int. J. Pediatr. Otorhinolaryngol..

[13] Bluestone C.D., Klein J.O. (2007). Otitis Media in Infants and Children.

[14] Digilio M.C., Pacifico C., Tieri L., Marino B., Giannotti A., Dallapiccola B. (1999). Audiological findings in patients with microdeletion 22q11 (di George/velocardiofacial syndrome). Br. J. Audiol..

[15] Rosenfeld R.M., Culpepper L., Doyle K.J., Grundfast K.M., Hoberman A., Kenna M.A., Lieberthal A.S., Mahoney M., Wahl R.A., Woods C.R. (2004). Clinical practice guideline: Otitis media with effusion. Otolaryngol. Head. Neck Surg..

[16] Paradise J.L., Rockette H.E., Colborn D.K., Bernard B.S., Smith C.G., Kurs-Lasky M., Janosky J.E. (1997). Otitis media in 2253 Pittsburgh-area infants: prevalence and risk factors during the first two years of life. Pediatrics.

[17] Gates G.A., Klein J.O., Lim D.J., Mogi G., Ogra P.L., Pararella M.M., Paradise J.L., Tos M. (2002). Recent advances in otitis media. 1. Definitions, terminology, and classification of otitis media. Ann. Otol. Rhinol. Laryngol. Suppl..

[18] Bhutta M.F. (2014). Epidemiology and pathogenesis of otitis media: construction of a phenotype landscape. Audiol. Neurootol..

[19] Salomonsen R.L., Hermansson A., Cayé-Thomasen P. (2010). Ossicular bone modeling in acute otitis media. Otol. Neurotol..

[20] Lannigan F.J., O'Higgins P., Mcphie P. (1993). The cellular mechanism of ossicular erosion in chronic suppurative otitis media. J. Laryngol. Otol..

[21] Lima K., Følling I., Eiklid K.L., Natvig S., Abrahamsen T.G. (2010). Age-dependent clinical problems in a Norwegian national survey of patients with the 22q11.2 deletion syndrome. Eur. J. Pediatr..

[22] Uhari M., Mäntysaari K., Niemelä M. (1996). A meta-analytic review of the risk factors for acute otitis media. Clin. Infect. Dis..

[23] Preciado D., Kuo E., Ashktorab S., Manes P., Rose M. (2010). Cigarette smoke activates NFκB-mediated Tnf-α release from mouse middle ear cells. Laryngoscope.

[24] Bluestone C.D. (1998). Epidemiology and pathogenesis of chronic suppurative otitis media: implications for prevention and treatment. Int. J. Pediatr. Otorhinolaryngol..

[25] Bluestone C.D., Doyle W.J. (1988). Anatomy and physiology of Eustachian tube and middle ear related to otitis media. J. Allergy Clin. Immunol..

[26] Graves G.O., Edwards L.F. (1944). The Eustachian tube: a review of its descriptive, microscopic and clinical anatomy. Arch. Otolaryngol. Head Neck Surg..

[27] Bluestone C.D. (1983). Eustachian tube function: physiology, pathophysiology, and role of allergy in pathogenesis of otitis media. J. Allergy Clin. Immunol..

[28] Albiin N., Hellström S., Salén B., Stenfors L.E., Söderberg O. (1983). The anatomy of the Eustachian tube in the rat: a macro- and microscopical study. Anat. Rec..

[29] Bylander-Groth A., Stenström C. (1998). Eustachian tube function and otitis media in children. Ear. Nose. Throat J..

[30] Cober M.P., Johnson C.E. (2005). Otitis media: review of the 2004 treatment guidelines. Ann. Pharmacother..

[31] Takasaki K., Takahashi H., Miyamoto I., Yoshida H., Yamamoto-Fukuda T., Enatsu K., Kumagami H. (2007). Measurement of angle and length of the Eustachian tube on computed tomography using the multiplanar reconstruction technique. Laryngoscope.

[32] Casselbrant M.L., Mandel E.M., Fall P.A., Rockette H.E., Kurs-Lasky M., Bluestone C.D., Ferrell R.E. (1999). The heritability of otitis media: a twin and triplet study. JAMA.

[33] Galili N., Baldwin H.S., Lund J., Reeves R., Gong W., Wang Z., Roe B.A., Emanuel B.S., Nayak S., Mickanin C. (1997). A region of mouse chromosome 16 is syntenic to the DiGeorge, velocardiofacial syndrome minimal critical region. Genome Res..

[34] Sutherland H.F., Kim U.J., Scambler P.J. (1998). Cloning and comparative mapping of the DiGeorge syndrome critical region in the mouse. Genomics.

[35] Paylor R., McIlwain K.L., McAninch R., Nellis A., Yuva-Paylor L.A., Baldini A., Lindsay E.A. (2001). Mice deleted for the DiGeorge/velocardiofacial syndrome region show abnormal sensorimotor gating and learning and memory impairments. Hum. Mol. Genet..

[36] Paylor R., Glaser B., Mupo A., Ataliotis P., Spencer C., Sobotka A., Sparks C., Choi C.-H., Oghalai J., Curran S. (2006). Tbx1 haploinsufficiency is linked to behavioral disorders in mice and humans: implications for 22q11 deletion syndrome. Proc. Natl Acad. Sci. USA.

[37] Lindsay E., Botta A., Jurecic V., Carattini-Rivera S., Cheah Y.C., Rosenblatt H.M., Bradley A., Baldini A. (1999). Congenital heart disease in mice deficient for the DiGeorge syndrome region. Nature.

[38] Fuchs J.C., Zinnamon F.A., Taylor R.R., Ivins S., Scambler P.J., Forge A., Tucker A.S., Linden J.F. (2013). Hearing loss in a mouse model of 22q11.2 deletion syndrome. PLoS One.

[39] Funke B., Epstein J.A., Kochilas L.K., Lu M.M., Pandita R.K., Liao J., Bauerndistel R., Schüler T., Schorle H., Brown M.C. (2001). Mice overexpressing genes from the 22q11 region deleted in velo-cardio-facial syndrome/DiGeorge syndrome have middle and inner ear defects. Hum. Mol. Genet..

[40] Jerome L.A., Papaioannou V.E. (2001). DiGeorge syndrome phenotype in mice mutant for the T-box gene, Tbx1. Nat. Genet..

[41] Liao J., Kochilas L., Nowotschin S., Arnold J.S., Aggarwal V.S., Epstein J.A., Brown M.C., Adams J., Morrow B.E. (2004). Full spectrum of malformations in velo-cardio-facial syndrome/DiGeorge syndrome mouse models by altering Tbx1 dosage. Hum. Mol. Genet..

[42] Rye M.S., Bhutta M.F., Cheeseman M.T., Burgner D., Blackwell J.M., Brown S.D.M., Jamieson S.E. (2011). Unraveling the genetics of otitis media: from mouse to human and back again. Mamm. Genome.

[43] MacArthur C.J., Hefeneider S.H., Kempton J.B., Trune D.R. (2006). C3H/HeJ mouse model for spontaneous chronic otitis media. Laryngoscope.

[44] Rivkin A.Z., Palacios S.D., Pak K., Bennett T., Ryan A.F. (2005). The role of Fas-mediated apoptosis in otitis media: observations in the lpr/lpr mouse. Hear. Res..

[45] Yang A., Walker N., Bronson R., Kaghad M., Oosterwegel M., Bonnin J., Vagner C., Bonnet H., Dikkes P., Sharpe A. (2000). p73-deficient mice have neurological, pheromonal and inflammatory defects but lack spontaneous tumours. Nature.

[46] Hilton J.M., Lewis M.A., Grati M., Ingham N., Pearson S., Laskowski R.A., Adams D.J., Steel K.P. (2011). Exome sequencing identifies a missense mutation in Isl1 associated with low penetrance otitis media in dearisch mice. Genome Biol..

[47] Cheeseman M.T., Tyrer H.E., Williams D., Hough T.A., Pathak P., Romero M.R., Hilton H., Bali S., Parker A., Vizor L. (2011). HIF-VEGF pathways are critical for chronic otitis media in Junbo and Jeff mouse mutants. PLoS Genet..

[48] Danielian P.S., Bender Kim C.F., Caron A.M., Vasile E., Bronson R.T., Lees J.A. (2007). E2f4 is required for normal development of the airway epithelium. Dev. Biol..

[49] Depreux F.F.S., Darrow K., Conner D.A., Eavey R.D., Liberman M.C., Seidman C.E., Seidman J.G. (2008). Eya4-deficient mice are a model for heritable otitis media. J. Clin. Invest..

[50] Noben-Trauth K., Latoche J.R. (2011). Ectopic mineralization in the middle ear and chronic otitis media with effusion caused by RPL38 deficiency in the Tail-short (Ts) mouse. J. Biol. Chem..

[51] Yang B., Tian C., Zhang Z.G., Han F.C., Azem R., Yu H., Zheng Y., Jin G., Arnold J.E., Zheng Q.Y. (2011). Sh3pxd2b mice are a model for craniofacial dysmorphology and otitis media. PLoS One.

[52] Tian C., Yu H., Yang B., Han F., Zheng Y., Bartels C.F., Schelling D., Arnold J.E., Scacheri P.C., Zheng Q.Y. (2012). Otitis media in a new mouse model for CHARGE syndrome with a deletion in the Chd7 gene. PLoS One.

[53] Zhang Y., Yu H., Xu M., Han F., Tian C., Kim S. (2012). Pathological features in the Lmna Dhe/ϩ mutant mouse provide a novel model of human otitis media and laminopathies.

[54] Hardisty R.E., Erven A., Logan K., Morse S., Guionaud S., Sancho–Oliver S., Jackie Hunter A., Brown S.D.M., Steel K.P. (2003). The deaf mouse mutant Jeff (Jf) is a single gene model of Otitis media. J. Assoc. Res. Otolaryngol..

[55] Hardisty-Hughes R.E., Tateossian H., Morse S.A., Romero M.R., Middleton A., Tymowska-Lalanne Z., Hunter A.J., Cheeseman M., Brown S.D.M. (2006). A mutation in the F-box gene, Fbxo11, causes otitis media in the Jeff mouse. Hum. Mol. Genet..

[56] Segade F., Daly K.A., Allred D., Hicks P.J., Cox M., Brown M., Hardisty-Hughes R.E., Brown S.D.M., Rich S.S., Bowden D.W. (2006). Association of the FBXO11 gene with chronic otitis media with effusion and recurrent otitis media: the Minnesota COME/ROM Family Study. Arch. Otolaryngol. Head. Neck Surg..

[57] Bhutta M.F. (2012). Mouse models of otitis media: strengths and limitations. Otolaryngol. Head. Neck Surg..

[58] Park K., Ueno K., Lim D.J. (1992). Developmental anatomy of the Eustachian tube and middle ear in mice. Am. J. Otolaryngol..

[59] Vitelli F., Morishima M., Taddei I., Lindsay E.A., Baldini A. (2002). Tbx1 mutation causes multiple cardiovascular defects and disrupts neural crest and cranial nerve migratory pathways. Hum. Mol. Genet..

[60] Thompson H., Tucker A.S. (2013). Dual origin of the epithelium of the mammalian middle ear. Science.

[61] Chapman D.L., Garvey N., Hancock S., Alexiou M., Agulnik S.I., Gibson-Brown J.J., Cebra-Thomas J., Bollag R.J., Silver L.M., Papaioannou V.E. (1996). Expression of the T-box family genes, Tbx1-Tbx5, during early mouse development. Dev. Dyn..

[62] Moraes F., Nóvoa A., Jerome-Majewska L.A., Papaioannou V.E., Mallo M. (2005). Tbx1 is required for proper neural crest migration and to stabilize spatial patterns during middle and inner ear development. Mech. Dev..

[63] Kong P., Racedo S.E., Macchiarulo S., Hu Z., Carpenter C., Guo T., Wang T., Zheng D., Morrow B.E. (2014). Tbx1 is required autonomously for cell survival and fate in the pharyngeal core mesoderm to form the muscles of mastication. Hum. Mol. Genet..

[64] Richter C.A., Amin S., Linden J., Dixon J., Dixon M.J., Tucker A.S. (2010). Defects in middle ear cavitation cause conductive hearing loss in the Tcof1 mutant mouse. Hum. Mol. Genet..

[65] Yeger H., Minaker E., Charles D., Rubin A., Sturgess J.M. (1988). Abnormalities of cilia in the middle ear in chronic otitis media. Ann. Otol. Rhinol. Laryngol..

[66] Lindsay E.A., Vitelli F., Su H., Morishima M., Huynh T., Pramparo T., Jurecic V., Ogunrinu G., Sutherland H.F., Scambler P.J. (2001). Tbx1 haploinsufficieny in the DiGeorge syndrome region causes aortic arch defects in mice. Nature.

[67] Arnold J.S., Werling U., Braunstein E.M., Liao J., Nowotschin S., Edelmann W., Hebert J.M., Morrow B.E. (2006). Inactivation of Tbx1 in the pharyngeal endoderm results in 22q11DS malformations. Development.

[68] McDonald M.H., Hoffman M.R., Gentry L.R., Jiang J.J. (2012). New insights into mechanism of Eustachian tube ventilation based on cine computed tomography images. Eur. Arch. Otorhinolaryngol..

[69] Sudo M., Sando I., Suzuki C. (1998). Three-dimensional reconstruction and measurement study of human Eustachian tube structures: a hypothesis of Eustachian tube function. Ann. Otol. Rhinol. Laryngol..

[70] Hecht C.S., Gannon P.J., Eden A.R. (1993). Motor innervation of the Eustachian tube muscles in the guinea pig. Laryngoscope.

[71] Honjo I., Okazaki N., Ushiro K., Kumazawa T. (1980). Cineradiographic analysis of Eustachian tube function. Experimental study. Ann. Otol. Rhinol. Laryngol..

[72] Kelly R.G., Jerome-Majewska L.A., Papaioannou V.E. (2004). The del22q11.2 candidate gene Tbx1 regulates branchiomeric myogenesis. Hum. Mol. Genet..

[73] Chang K.H., Jun B.C., Jeon E.J., Park Y.-S. (2013). Functional evaluation of paratubal muscles using electromyography in patients with chronic unilateral tubal dysfunction. Eur. Arch. Otorhinolaryngol..

[74] Finkelstein Y., Talmi Y.P., Nachmani A., Hauben D.J., Zohar Y. (1990). Levator veli palatini muscle and Eustachian tube function. Plast. Reconstr. Surg..

[75] Ishijima K., Sando I., Balaban C.D., Miura M., Takasaki K. (2002). Functional anatomy of levator veli palatini muscle and tensor veli palatini muscle in association with Eustachian tube cartilage. Ann. Otol. Rhinol. Laryngol..

[76] Garg V., Yamagishi C., Hu T., Kathiriya I.S., Yamagishi H., Srivastava D. (2001). Tbx1, a DiGeorge syndrome candidate gene, is regulated by sonic hedgehog during pharyngeal arch development. Dev. Biol..

[77] Xu H., Cerrato F., Baldini A. (2005). Timed mutation and cell-fate mapping reveal reiterated roles of Tbx1 during embryogenesis, and a crucial function during segmentation of the *pharyngeal* system via regulation of endoderm expansion. Development.

[78] Calmont A., Ivins S., Van Bueren K.L., Papangeli I., Kyriakopoulou V., Andrews W.D., Martin J.F., Moon A.M., Illingworth E.A., Basson M.A. (2009). Tbx1 controls cardiac neural crest cell migration during arch artery development by regulating Gbx2 expression in the pharyngeal ectoderm. Development.

[79] Karpinski B.A., Maynard T.M., Fralish M.S., Nuwayhid S., Zohn I.E., Moody S.A., LaMantia A.-S. (2014). Dysphagia and disrupted cranial nerve development in a mouse model of DiGeorge (22q11) deletion syndrome. Dis. Model. Mech..

[80] Guris D.L., Duester G., Papaioannou V.E., Imamoto A. (2006). Dose-dependent interaction of Tbx1 and Crkl and locally aberrant RA signaling in a model of del22q11 syndrome. Dev. Cell.

[81] Ivins S., Lammerts van Beuren K., Roberts C., James C., Lindsay E., Baldini A., Ataliotis P., Scambler P.J. (2005). Microarray analysis detects differentially expressed genes in the pharyngeal region of mice lacking Tbx1. Dev. Biol..

[82] Reading R. (2011). Grommets (ventilation tubes) for hearing loss associated with otitis media with effusion in children. Child. Care. Health Dev..

[83] Yaman H., Yilmaz S., Alkan N., Subasi B., Guclu E., Ozturk O. (2010). Shepard grommet tympanostomy tube complications in children with chronic otitis media with effusion. Eur. Arch. Otorhinolaryngol..

[84] Tavernier L., Chobaut J. (2006). Eustachian tube rehabilitation therapy : indications, techniques, and results. Fr ORL..

[85] Kim J., Sapienza C.M. (2005). Implications of expiratory muscle strength training for rehabilitation of the elderly: tutorial. J. Rehabil. Res. Dev..

[86] Guo C., Sun Y., Zhou B., Adam R.M., Li X., Pu W.T., Morrow B.E., Moon A., Li X. (2011). A Tbx1-Six1/Eya1-Fgf8 genetic pathway controls mammalian cardiovascular and craniofacial morphogenesis. J. Clin. Invest..

[87] Grifone R., Laclef C., Spitz F., Demignon J., Guidotti J., Kawakami K., Xu P., Kelly R., Basil J., Daegelen D. (2004). Six1 and Eya1 expression can reprogram adult muscle from the slow-twitch phenotype into the fast-twitch phenotype Six1 and Eya1 expression can reprogram adult muscle from the slow-twitch phenotype into the fast-twitch phenotype. Mol. Cell Biol..

[88] Xu P.-X., Zheng W., Laclef C., Maire P., Maas R.L., Peters H., Xu X. (2002). Eya1 is required for the morphogenesis of mammalian thymus, parathyroid and thyroid. Development.

[89] Worley G.A., Vats A., Harcourt J., Albert D.M. (1999). Bilateral congenital cholesteatoma in branchio-oto-renal syndrome. J. Laryngol. Otol..

[90] Zim S., Schelper R., Kellman R., Tatum S., Ploutz-Snyder R., Shprintzen R. (2003). Thickness and histologic and histochemical properties of the superior pharyngeal constrictor muscle in velocardiofacial syndrome. Arch. Facial Plast. Surg..

[91] Grifone R., Jarry T., Dandonneau M., Grenier J., Duprez D., Kelly R.G. (2008). Properties of branchiomeric and somite-derived muscle development in Tbx1 mutant embryos. Dev. Dyn..

[92] Weinberg E.S., Allende M.L., Kelly C.S., Abdelhamid A., Murakami T., Andermann P., Doerre O.G., Grunwald D.J., Riggleman B. (1996). Developmental regulation of zebrafish MyoD in wild-type, no tail and spadetail embryos. Development.

[93] Wilkinson D.G. (1998). In Situ Hybridization: A Practical Approach.

