# Supplementary material for: A defect in early myogenesis causes Otitis media in two mouse models of 22q11.2 Deletion Syndrome
Source: Hum Mol Genet. 2014 Dec 1;24(7):1869–82. doi: 10.1093/hmg/ddu604 (PMC4355021; doi:10.1093/hmg/ddu604)
Supplement: Supplementary Data [file supp_24_7_1869__index.html]

A defect in early myogenesis causes Otitis media in two mouse models of 22q11.2 deletion syndrome — A defect in early myogenesis causes Otitis media in two mouse models of 22q11.2 Deletion Syndrome — A defect in early myogenesis causes Otitis media in two mouse models of 22q11.2 Deletion Syndrome — Supplementary Data 

# A defect in early myogenesis causes Otitis media in two mouse models of 22q11.2 Deletion Syndrome

## Supplementary Data

Supplementary Data

**Files in this Data Supplement:**

- Supplementary Data - Docx file
