# Supplementary material for: A defect in early myogenesis causes Otitis media in two mouse models of 22q11.2 Deletion Syndrome
Source: Hum Mol Genet. 2014 Dec 1;24(7):1869–82. doi: 10.1093/hmg/ddu604 (PMC4355021; doi:10.1093/hmg/ddu604)
Supplement: Supplementary Data [file supp_ddu604_ddu604supp.docx]

**Supplementary Figure 1**

A) 3D-reconstruction of an adult WT skull. Red box indicates the left auditory bulla. B, C) Rendered MicroCT scans of the left auditory bulla. The orange line is indicating the length (B) and width (C) measured for analyzing auditory bulla morphology and size. D,E) Two litters containing 5 WT mice and 8 *Df1/+* mice between the ages of 11.5 to 18.5 weeks and varying degrees of the inflammation in either one or both ears were analysed. Comparing both length (D) and width (E) of the auditory bullae in *Df1/+* mice to WT littermates, we found a significant reduction of both values (p=0.0002 and p<0.0001 respectively). Since we measured the bulla size in both ears of each animal, we were also able to compare bulla size between ears with and without inflammation; we found no significant differences in bulla length or width between inflamed and un-inflamed ears (p=0.2293 for length and p=0.3007 for width). Statistical analysis was performed using student t-test. Scale bar: A,B,C 1mm.

**Supplementary Figure 2**

A) Masson’s Trichrome stained frontal sections of juvenile (P18) control mice showing the tensor tympani muscle (mTT, yellow) running along the ET and inserting into the tubal cartilage (TC) and nasopharynx (NP). B) In *Tbx1^+/-^* mice, the mTT appears smaller. C) Measurements of the mTT showing a significantly smaller muscle volume in *Tbx1^+/-^* mice (n=10) compared to control littermates (n=3, p=0.014). D) Graph displaying the correlation between Otitis media and mTT volume. We found no significantly reduced muscle size in ears with signs of an inflammation (n=4) compared to uninflamed ears (n=4, p=0.3429). Statistical analysis was performed using non-parametric Mann-Whitney test. Scale bar: 250µm.


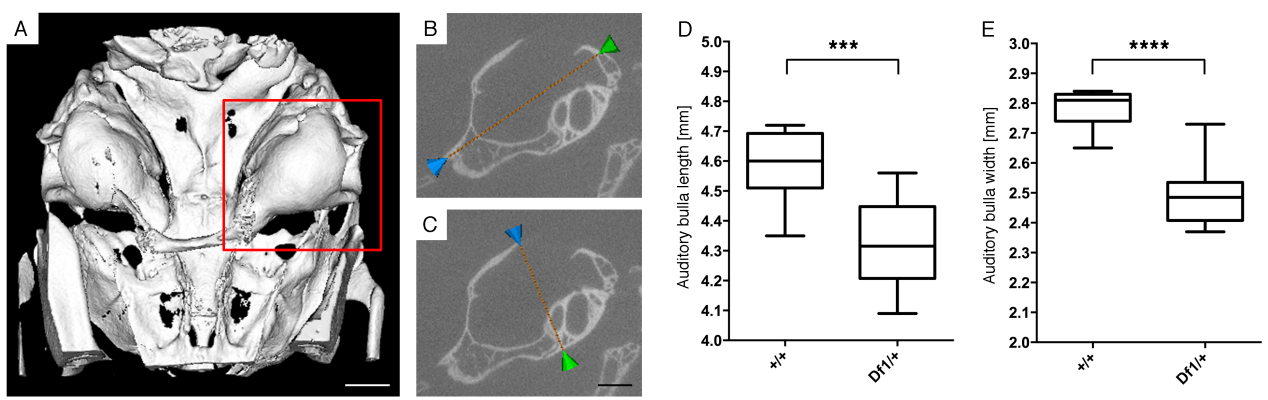


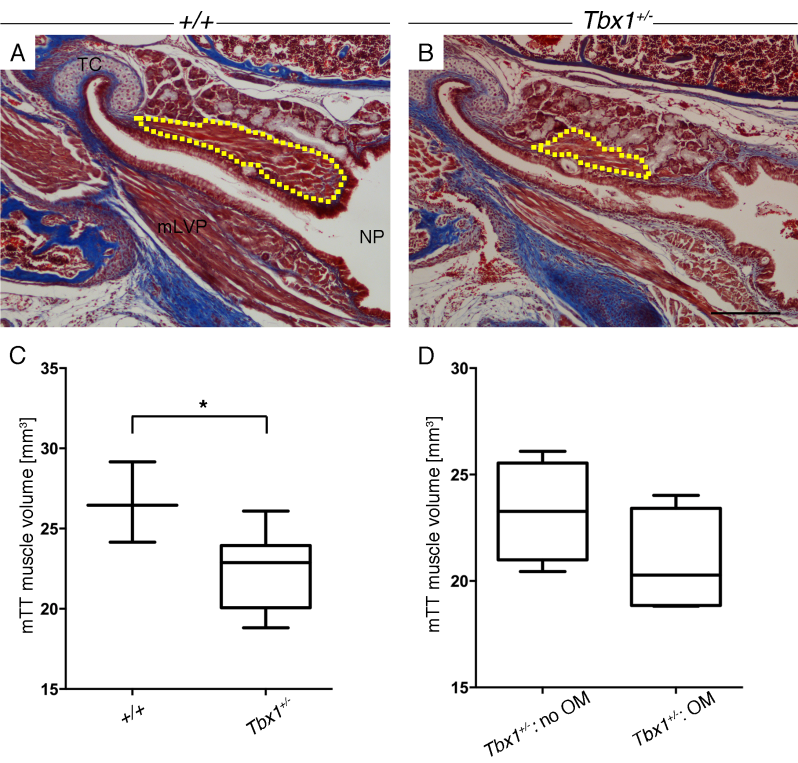


**Supplementary Table 1**

Table displaying the presence of Otitis media and the muscle sizes of the tensor tympani (mTT) and levator veli palateni muscle (mLVP) of one P18 litter (2 control mice and 5 *Tbx1^+/-^* mice). The ear with blood infiltrate was not used in the subsequent analysis as the status of OM could not be verified.

**Supplementary Table 1**

|  | **left muscle** | | **right muscle** | |
| --- | --- | --- | --- | --- |
| **mTT** | **volume [mm3]** | **OM (Y/N)** | **volume [mm3]** | **OM (Y/N)** |
| *+/+* (1) | 29.16 | N | 24.15 | N |
| *+/+* (2) | 26.46 | N | n/a | N |
| *Tbx1^+/-^* (1) | 18.82 | Y | 22.64 | N |
| *Tbx1^+/-^* (2) | 23.6 | blood infiltrate | 23.12 | blood infiltrate |
| *Tbx1^+/-^* (3) | 20.44 | N | 18.95 | Y |
| *Tbx1^+/-^* (4) | 24.02 | Y | 21.61 | Y |
| *Tbx1^+/-^* (5) | 26.09 | N | 23.91 | N |
|  |  |  |  |  |
|  | **left muscle** | | **right muscle** | |
| **mLVP** | **volume [mm3]** | **OM (Y/N)** | **volume [mm3]** | **OM (Y/N)** |
| *+/+* (1) | 28.93 | N | 28.17 | N |
| *+/+* (2) | 24.02 | N | 25.47 | N |
| *Tbx1^+/-^* (1) | 14.45 | Y | 16.26 | N |
| *Tbx1^+/-^* (2) | 13.81 | blood infiltrate | 13 | blood infiltrate |
| *Tbx1^+/-^* (3) | 18.36 | N | 13.66 | Y |
| *Tbx1^+/-^* (4) | 13.46 | Y | 14.55 | Y |
| *Tbx1^+/-^* (5) | 16.02 | N | 15.01 | N |
